# Impact of COVID-19 on Basic Life Support Training Among Medical Students: An Experimental Study

**DOI:** 10.7759/cureus.23775

**Published:** 2022-04-03

**Authors:** Narayanan Rajaram, Harini Krishna, Ritu Singh, Anil K Narayan

**Affiliations:** 1 Anesthesiology and Critical Care, Andaman & Nicobar Islands Institute of Medical Sciences (ANIIMS), Port Blair, IND; 2 Anesthesiology and Critical Care, Chandramma Dayananda Sagar Institute of Medical Education and Research, Kanakapura, IND; 3 Paediatrics, Andaman & Nicobar Islands Institute of Medical Sciences (ANIIMS), Port Blair, IND

**Keywords:** competency based medical education, drscab, doap method, foundation course, hands-on experience, irc guidelines, self-directed learning, covid – 19, medical students, bls training

## Abstract

Aims and objectives: Sudden cardiac death (SCD) is the most common cause of mortality worldwide. Bystander cardiopulmonary resuscitation (CPR) improves the victim's outcome, especially when the response time for advanced life support is prolonged. We performed a study to estimate the difference in knowledge among first-year medical students after basic life support (BLS) training (part of their foundation course) before and during the novel COVID-19 pandemic.

Materials and methods: We recruited first-year medical college students (batch of 2019-20: pre-COVID group - P and batch of 2020-21: COVID-19 era group - C) who were undergoing BLS training for the first time and consented to this study. Since the training was delayed and affected by COVID-19 for the batch of 2020-21, their training duration was shorter with more usage of audiovisual aids. The difference in the change in knowledge (by a questionnaire with 10 questions of one mark each) after training by the two methods was analysed. Analysis of variance, Wilcoxon signed-rank test, Mann-Whitney U test, and chi-square tests was used as applicable to compare the groups, and p-value <0.05 was considered significant. The results are analysed by IBM SPSS version 20.0 software (SPSS Inc, Chicago, IL, USA).

Results: The median (inter-quartile range) marks in group P (89 students) in the pre-test was 3 (4-2) and in the post-test was 6 (7-5) (out of 10). The corresponding marks in group C (112 students) in the pre-test were 3 (4-2) and in post-test was 7 (8-6). The knowledge improvement in group C was more with all the three changes being significant (p=0.0001). In group C, females had more improvement than males (p=0.0001).

Conclusion: We found a significant increase in the improvement of the knowledge after the BLS training in group C compared to group P. In group C, the improvement was better in females (59% increase in mean marks versus 22% in males).

## Introduction

Sudden cardiac death (SCD) is the leading cause of mortality worldwide, with the incidence in western countries being approximately 50 to 100 per 100,000 in the general population [1]. SCD accounts for 10.3% of all the deaths in South India and the victims are 5-8 years younger than their western counterparts [2]. Bystander cardiopulmonary resuscitation (CPR) improves the outcome of BLS especially when the initiation of advanced life support is delayed [3]. Basic life support (BLS) training has been a neglected area for the training of medical students and the knowledge and awareness of BLS among medical students are very poor as per the results of several studies and surveys conducted in the past [4-6]. Implementation of a longitudinal CPR elective improved resuscitation science education for medical students and fostered increased community CPR training [7]. incorporation of a BLS course, including CPR learning procedures in the university curriculum, with regular reassessments, would increase the knowledge and application of CPR skills among students for saving people's life [8-9]. 

The revised curriculum for undergraduate medical trainees in India now includes BLS training as an integral part of the foundation course for first-year medical students [10]. The lack of sufficient practical training sessions during the COVID-19 pandemic could have hurt the training for this vital skill.

We could not find any studies in the literature regarding the effect of the COVID-19 pandemic on the outcome after BLS training for first-year medical students. Hence, we performed a study to estimate the difference in knowledge among first-year medical students after BLS training and to compare this outcome before and during the COVID-19 pandemic. The secondary objective was to study two modes of BLS training - the first mode was largely offline teaching with hands-on experience and the other one was a mixed way of teaching involving online and offline methods without hands-on experience.

## Materials and methods

After obtaining the approval of the Institutional Ethics Committee, we recruited first-year medical college students who had not undergone BLS training previously and consented to this study (batch of 2019-20 and batch of 2020-21). They had to undergo BLS training as a part of their skills module during their foundation course. Students who gave informed consent were included in the study (refer to Participant Information Sheet and Consent Form in the Appendices).

The first batch underwent the BLS training as per National Medical Council (NMC) norms which were spread out over five days. Eighty-nine students took part in the study. On the first day, we gave a questionnaire (pre-test) containing 10 questions to test the knowledge component of BLS (refer to Pre Test - Questionnaire in the Appendices). This was followed by an audio-visual presentation about the theoretical aspects and a video demonstration of BLS. This was followed by the physical demonstration of the BLS sequence using the Laerdal whole-body mannequin (Laerdal, Stavanger, Norway) by the anaesthesiology faculty. From the second through the fifth day, they were divided into five groups and all of them were made to perform BLS sequence in pairs. After this, on the fifth day, they were again given the questionnaire (post-test containing 10 questions) along with their feedback regarding the training (refer to Post Test - Questionnaire in the Appendices). All the participating students were asked to write their name and roll numbers on the mark sheets which was then coded by a clerk. This group is denoted as the pre-COVID group (P).

Since the training was delayed and affected by COVID-19 for the batch of 2020-21, there was a shorter orientation programme for them after the crisis reduced in severity. Informed consent was obtained from the medical students who were willing to participate in the study. A total of 112 students took part in the study and they were told to write their names and roll numbers which were again coded. We conducted a pre-test to test the knowledge regarding BLS using the same questionnaire of 10 questions at the end of their first day of the orientation programme before any training in BLS was given. We sent PowerPoint presentations regarding BLS online to the medical students. The content included theoretical aspects, practical demonstration of BLS, links to free source articles - Adult Basic and Advanced Life Support: 2020 American Heart Association Guidelines for Cardiopulmonary Resuscitation and Emergency Cardiovascular Care and Indian Resuscitation Council guidelines (an initiative by ISA - www.cprindia.in) [10-11]. Students were advised to learn only BLS and compression-only life support (COLS) modules through these links. We advised them to go through the videos repeatedly to get well versed with the sequence after taking an hour of a class of Powerpoint presentation and demonstration of the same with a mannequin by the anesthesiology faculty, following strict COVID-19 protocol. There was no hands-on training by the medical students and their close interaction with the faculty was avoided. At the end of the training, they were given a post-test similar to the previous batch along with their feedback. Again, the set of papers was coded and was sent for marking by a clerk. This group is denoted as the COVID-19 group (C) (online and offline teaching without hands-on experience).

A master chart was prepared using Microsoft Excel. Analysis of variance, Kruskal-Wallis test, Mann-Whitney U test, Wilcoxon signed-ranks test and chi-square tests were used as applicable to compare the groups; p-value <0.05 was considered significant. The results are analysed by IBM SPSS version 20.0 software (SPSS Inc, Chicago, IL, USA).

The pre-test and post-test of the 2019-20 batch (group P) were analysed for improvement in knowledge post the BLS training which was done after the offline classes with hands-on experience. The pre-test and post-test of the 2020-21 batch (group C) were analysed for improvement in knowledge after the BLS training was done after the online and offline classes in the COVID-19 group (C). The difference in change in knowledge after the training by the above two methods was also analysed which was our primary objective.

## Results

All the 89 students in group P and 112 students in group C participated throughout the study and their data was used for final analysis. 

The 2019 (pre-C0VID) batch (group P) had 89 medical students with 48 male and 41 female students (Table 1)

**Table 1:** Demographics of 2019 batch (Group P)

Sex	Frequency	Percent
Female	48	53.9
Male	41	46.1
Total	89	100.0

The 2020 batch had 112 (group C) medical students with 73 female and 39 male students (Table 2).

**Table 2:** Demographics of 2020 batch (Group C)

Sex	Frequency	Percent
Female	73	65.2
Male	39	34.8
Total	112	100.0

The baseline characteristics in both the groups did not change significantly, all being first-year medical students; the gender difference between the two groups was not statistically significant (p=0.106) as seen in Table 3.

**Table 3:** Comparison of gender composition between 2019 and 2020 batch

Year	Gender		Total
	Female	Male	
2019 (Group P)	48(53.9%)	41(46.1%)	89
2020 (Group C)	73(65.2%)	39(34.8%)	112
Total	121(60.2%)	80(39.8%)	201

The marks for group P in the pre-test had a median of 3 (out of 10) with an interquartile range (IQR) of 2 (75th percentile at 4 and 25th percentile at 2 as can be seen in the table). The corresponding marks in the post-test were 6 (7-5). This was statistically significant when analysed by paired “t” test (P=0.0001) for the 89 medical students (Table 4).

**Table 4:** Knowledge change in 2019 batch (Group P)

Status	Median (IQR)	Wilcoxon Signed Ranks Test
Pre test	3.00 (4-2)	p=0.0001
Post test	6.00 (7-5)	

The marks for group P in the pre-test had a median of 3 (out of 10) with an IQR of 2 (4-2). The corresponding marks in the post-test were 7 (8-6) which again was statistically significant (p=0.0001) for the 112 medical students (Table 5).

**Table 5:** Knowledge change in 2020 batch (Group C)

Status	Median (IQR)	Wilcoxon Signed Ranks Test
Pre test	3.00 (4-2)	p=0.0001
Post test	7.00 (8-6)	

Also, there was a significant difference between the change in values between the two groups (p=0.0001) with more improvement in group C (median value (IQR) of 4 (5-3) as against the median value of 3 (4-1) in group P) (Table 6).

**Table 6:** Comparison between 2019 and 2020 batch

Difference in knowledge change	group	N	Median (IQR)	Mann Whitney U test
	2020 batch	112	4.00 (5-3)	p=0.0001
	2019 batch	89	3.00 (4-1)	

The median pre-test scores among the 48 females in group P was 3.00 (4.00-2.00) and it increased to 6.00 (6.75-4.00) and was statistically significant (p=0.0001). The corresponding scores among the 41 males were 3.00 (4.00-1.50) which increased to 6.00 (7.50-5.00) during the post-test. This was also statistically significant (p=0.0001) with more knowledge after the teaching (Table 7).

**Table 7:** Knowledge difference in males and females in the 2019 batch (Group P)

Sex	Status	N	Median (IQR)	Wilcoxon Signed Ranks Test
Female	Pre test	48	3.00 (4.00-2.00)	p=0.0001
	Post test	48	6.00 (6.75-4.00)	
Male	Pre test	41	3.00 (4.00-1.50)	p=0.0001
	Post test	41	6.00 (7.50-5.00)	

The median pre-test scores among the 73 females in group C was 3.00 (4-2) and it increased to 7 (8-6) in the post-test which was statistically significant (p=0.0001). The same among the 39 males was 3 (4-2) during the pre-test and 7 (8-6) during the post-test which was also statistically significant (p=0.0001) with more knowledge after the teaching (Table 8).

**Table 8:** Knowledge difference in males and females in the 2020 batch (Group C)

Sex	Status	N	Median (IQR)	Wilcoxon Signed Ranks Test
Female	Pre test	73	3.00 (4-2)	p=0.0001
	Post test	73	7.00 (8-6)	
Male	Pre test	39	3.00 (4-2)	p=0.0001
	Post test	39	7.00 (8-6)	

We also did a gender-specific comparison among the two groups for the knowledge change after the two different modes of teaching.

The median change in knowledge among the 48 females in group P was 3 (4-1) and among the 73 females in the group P was 4 (5-3). This change was statistically significant when analysed by the Mann-Whitney U test (p=0.0001) with more improvement in group C. The change in the knowledge among the 41 males in the group P was 4 (5-1.5) and among the 39 males in group C was 4 (6-3). In group C, the mean percentage of increase in the marks was 59% in females and 22% in males. The change in knowledge was not statistically significant when we compared the two groups of males (p=0.182) (Table 9).

**Table 9:** Gender difference in the knowledge change between two groups after training

Sex	Status	Group	N	Median (IQR)	Mann-Whitney U test
Female	Difference in knowledge change	2020	73	4.00 (5-3)	p=0.0001
		2019	48	3.00 (4-1)	
Male	Difference in knowledge change	2020	39	4.00 (6-3)	p=0.182
		2019	41	4.00 (5-1.5)	

This leads to the conclusion that males showed a similar increase in knowledge after either mode of BLS training while females exhibited more improvement in the COVID group (group C) than in the pre-COVID group (group P).

## Discussion

BLS training is now an integral part of the foundation course for the first-year medical students as per the Competency-Based Medical Education curriculum (CBME) laid down by the National Medical Commission (NMC, erstwhile Medical Council of India). The knowledge about BLS is not greatly different among first-year medical students when compared to laypeople even in developed countries [12-13]. Under the aegis of the Indian Society of Anaesthesiologists (ISA), the Indian Resuscitation Council (IRC) aims to train all the citizens of our country on BLS, especially school and college students. The curriculum to teach resuscitation in the foundation course is the one advocated by the IRC [14].

The proposed teaching-learning method for psychomotor skills of BLS is the demonstration-observation-assistance-performance (DOAP) technique as per CBME. BLS was taught in a simulated environment where the student watches videos to create interest and then has interactive lectures on BLS. The student then observes the skills on a mannequin which the trainer deconstructs, describes the steps and demonstrates the skill. The student is then assisted to perform on a mannequin to acquire these skills and then practices and performs individually on a mannequin, and finally, performs as a part of a team to attain this competency [10]. Due to the pandemic situation, BLS training had to be partially modified for the 2020 batch (C).

The BLS training, as per the new pattern, started in 2019 before the arrival of COVID-19 is as per the IRC guidelines. After COVID-19 started, certain modifications had to be made to comply with COVID-appropriate behaviour. This led to a more aggressive usage of audio-visual aids (PowerPoint slides, videos, etc.) in teaching which had the advantage of more active listening by the medical students thereby enhancing their self-directed learning (SDL). We studied the impact of this change in the BLS teaching method on the knowledge of medical students.

We found that there was a significant positive change in the improvement of knowledge about BLS in the COVID-19 batch (C) when compared to the improvement in the knowledge in the pre-COVID-19 batch (P). Though knowledge improvement was significant in both the batches, the improvement was much more in the C group. This was contrary to our hypothesis that BLS training with hands-on experience may lead to more improvement in knowledge change especially since we spent a lot of time in face-to-face interactive teaching. This may be due to more emphasis on self-directed learning in which, apart from our traditional way of teaching, we had also taught the students how to revise the course material repeatedly both by observing and practising the BLS sequence on their own, even though our face-to-face interaction time with them was much less. Shavit et al. noted that the review of videotapes coupled with feedback is the better teaching method for infant BLS skills acquisition for lay-lone-rescuers [15].

Peyton’s four-step approach as an instructional strategy to teach technical skills to learners was recommended by the European Resuscitation Council (ERC) for resuscitation training [16]. Originally constructed by Peyton for a 1:1 student-teacher ratio, it was adapted for resuscitation training more than ten years ago and is used in international courses for trauma or advanced life support course concepts. It is based on the following steps of instructions:

Demonstration: skill demonstration at normal speed without explanation; Deconstruction: repetition of the skill’s steps with elaborate explanation and encouragement of the learner to ask questions; Comprehension: learner’s explanation of the steps of the skill and demonstrator’s instruction on the correct performance. Necessary corrections from the demonstrator and repetitions of this step are performed until full understanding is achieved; Performance: the learner practices the skill under observation and receives feedback.

This four-step approach has been successfully modified in various settings with video and media assistance. The mode of teaching we use in CBME is a similar one called the DOAP method. It was to be modified in group (C) because of COVID-19. Both the modes of teaching were successful in increasing the knowledge about the BLS. We did not study the other components of outcome like skills, attitude, ethics, and communication. Issleib et al. observed that virtual reality usage in BLS training may be used for overall learning gain but still, skills are better learnt by the traditional way of seminar and training [17].

An important finding in our study was that the gender difference in knowledge improvement was found only in the COVID group (C) with more improvement among the females. This was statistically significant (p=0.0001).

Limitations of the study

We did not study the difference in the change in attitude, skills, ethics, or communication part of the BLS among the two groups. Teaching methods like virtual reality which may complement the traditional teaching methods were not used. The delivery of the knowledge contents was not affected by the pandemic, only the practice (hands-on practice) was affected. So, the assessment of knowledge alone may not be a true indicator of the impact of the pandemic [18]. We have studied the immediate knowledge change but the impact on long-term knowledge retention about BLS was not studied.

Controversies raised by the study

Differences in gender in the change in knowledge about BLS between the two groups studied needs to be evaluated by multi-centric randomised control trials. It also raises the question - does the improved knowledge change convert into an overall improvement in the outcome of the healthcare delivery among the target population?

## Conclusions

We found that there is a significant increase in the improvement in knowledge after the BLS training in first-year medical students in the COVID era (group C) when compared to the offline mode of training during the pre-COVID times with hands-on training (group P). Males showed a similar increase in knowledge after either mode of BLS training. The improvement was better in females than in males in the COVID era. We presume that it may be due to the better aptitude for the retention of audiovisual memory in females. We could not find any parallel study for this in the literature.

## Figures and Tables

**Figure 1:**
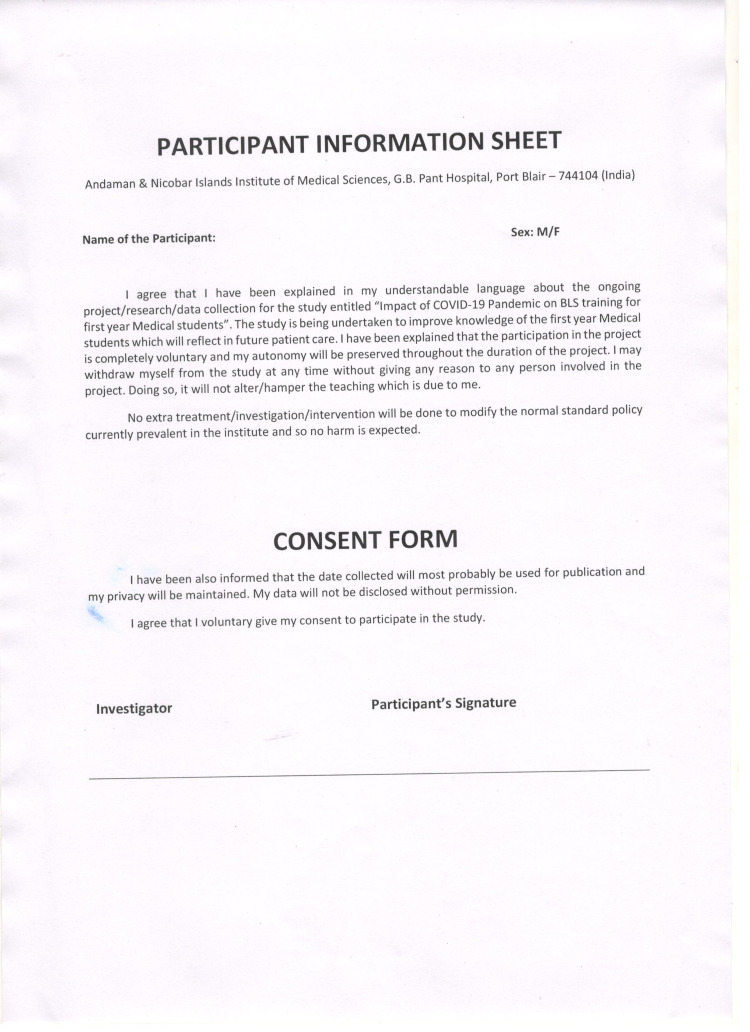
Participant Information Sheet and Consent Form

**Figure 2:**
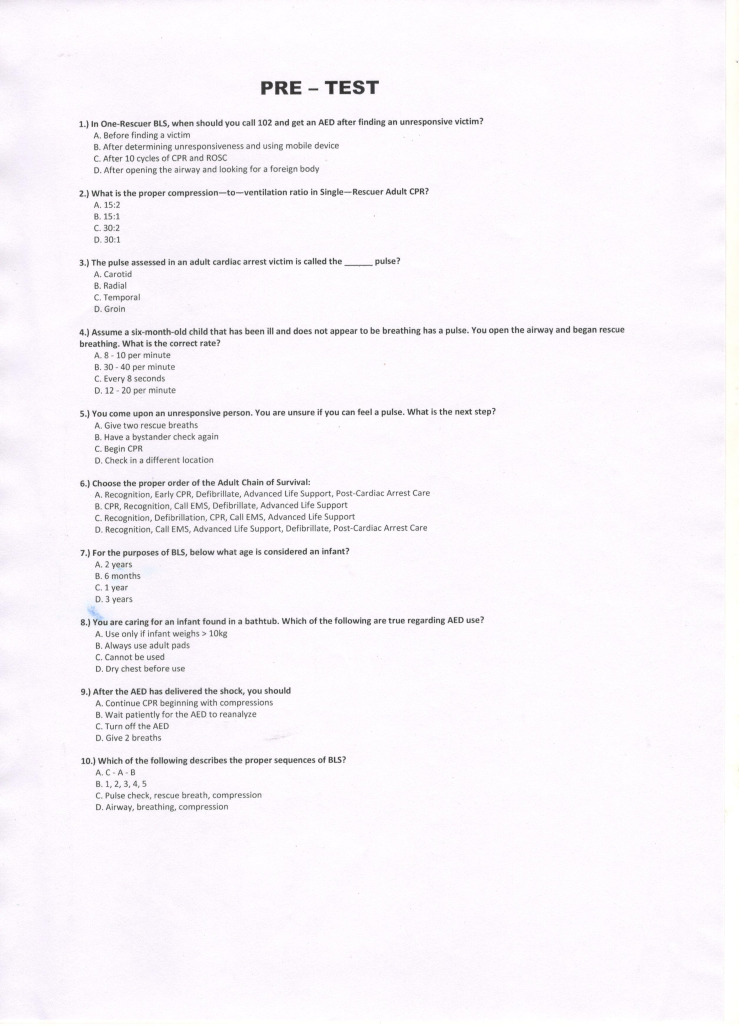
Pre Test - Questionnaire

**Figure 3:**
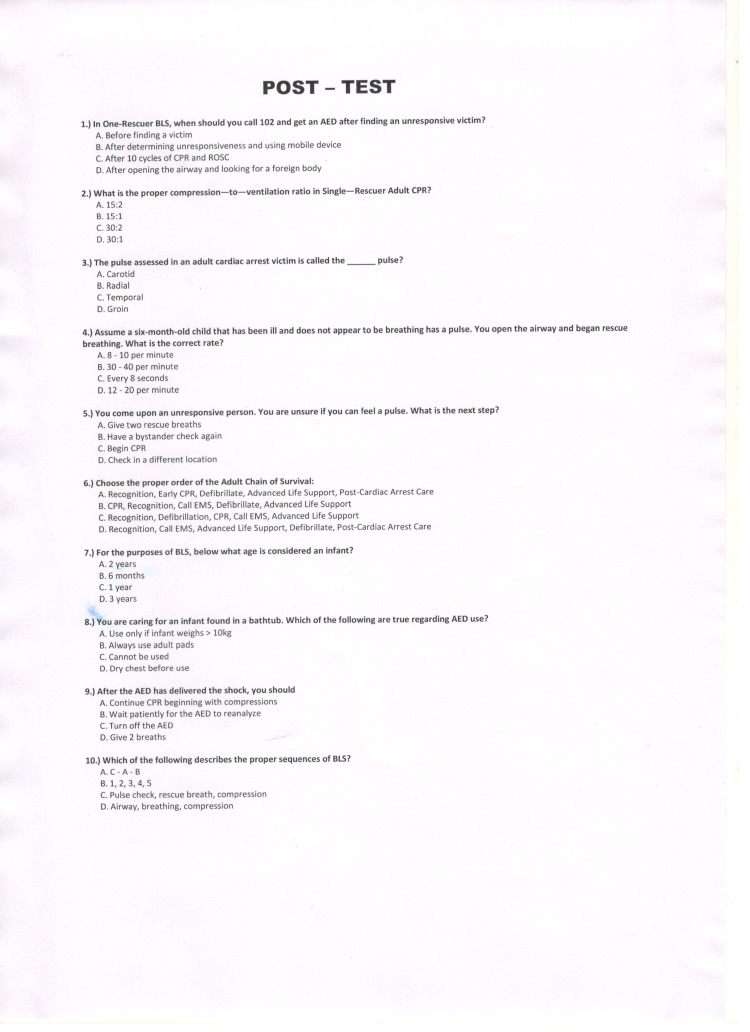
Post Test - Questionnaire
